# The South African Military Nursing Service

**Published:** 1918-06-29

**Authors:** 


### THE SOUTH AFRICAN MILITARY NURSING SERVICE.

The South African Military Nursing Service differs in essential particulars from the Nursing Services of the other Dominions. It was in 1915 that the first contingent of South African troops was sent over, and the first nurses arrived in October of that year. Members of the Service are recruited and chosen by Miss Nutt, Matron-in-Chief, Cape Town, and are all registered under the State Association. On their arrival in this country the sisters and nurses are placed under the authority of Miss Becher, R.R.C., Matron-in-Chief of Q.A.I.M.N.S.R., receiving the same pay and allowances as other members of this Service, and being in all respects subject to the same regulations. They wear, however, a distinctive uniform, of tussore colour, sisters having a dark-blue cape, while the staff nurses have a tussore cape edged with blue, Panama or dark-blue hats, and the springbok badge. They often stay at the Queen Mary Hostel for Nurses, where they are given hospitality until assigned to the hospital where they are to work. Many have been elected members of the (non-residential) Royal Club for Ladies from Beyond the Seas, Norfolk House. Quite a number of South African nurses have come over independently and have joined the Q.A.I.M.N.S.R., the T.F.N.S., or the B.R.C.N.S. There are altogether about 400 South African nurses and V.A.D. helpers now engaged in military nursing in this country and abroad.

The South African Hospital in Richmond Park, which was built and equipped by the South African Hospital and Comforts Fund, is almost entirely staffed by South African nurses, though the matron, Miss Jackson, is an Englishwoman. Originally built with 300 beds, with a view to expansion if found desirable, this fine institution has recently undergone a fresh extension, bringing its number of beds up to 1,200. It is mainly, though not exclusively, for the reception of members of the South African Forces. It is not merely a military hospital receiving the acutest cases, but also a curative and educational establishment, designed to raise the men from the fatalism and impaired will-power consequent on nerve-shock to the status of the bread-winner. The nursing staff consists of fourteen sisters, thirty-three staff nurses, and fifty-one V.A.D. probationers. Sisters Barber and Tilney, both of the S.A.M.N.S., have been awarded the Royal Red Cross for services rendered while in this institution. The No. 1 South African Hospital, B.E.F., is also staffed by the S.A.M.N.S., Mrs. Creagh, of Pretoria, being the matron.

The South African nurses have no Principal Matron of their own in this country, owing to the fact that they are mainly incorporated into other Services; but their individual interests are matters of intimate and personal concern. The Viscountess Gladstone, for the South African Comforts Committee, at their headquarters, 39 Grosvenor Place, keeps in touch with every nurse from the moment of her arrival. She receives them personally when they reach London, and sees to it that their leave before joining up is pleasantly spent. Should illness overtake any member of the Service or rest and change be recommended, arrangements are made for them such as they themselves prefer. A spirit of good will and friendship animates this whole organisation, which has the interests of South African men and women at heart, and reflects the real concern of South Africa in the sons and daughters she has sent over to represent her.

